# Case Report: Combination oral appliance therapy acute influence on cardiac electrophysiology and hemodynamics in OSA patient with paroxysmal atrial fibrillation

**DOI:** 10.3389/frsle.2025.1580381

**Published:** 2025-10-30

**Authors:** Preetam Schramm, Emet Schneiderman, Jason Hui, Zohre German, Ju Ying Lin

**Affiliations:** Department of Biomedical Sciences, Texas A&M University College of Dentistry, Dallas, TX, United States

**Keywords:** oral appliance, obstructive sleep apnea, premature atrial contraction, plethysmography, auto-adjusting positive airway pressure

## Abstract

**Background and objectives:**

Sleep apnea-related autonomic responses may increase cardiac arrhythmias. Ablation, cardioversion, and pharmacologic therapies for paroxysmal atrial fibrillation (AF) could benefit from adjunctive oral appliance therapy with a mouth shield (OAT+) compared to auto-adjusting positive airway pressure (APAP).

**Methods:**

A 67-year-old male with moderate obstructive sleep apnea (OSA), AF history, three ablations, and on Carvedilol (10 mg daily) underwent home sleep recordings with APAP and with OAT+ after 4 weeks. Randomly selected premature atrial contractions (PACs; n=20) and time-linked plethysmography waves from each intervention were compared.

**Results:**

OAT+ reduced the PAC index (-61.9%), cardiac conduction intervals (nR-R, *p* = 0.025; pre-PAC R-R, *p* = 0.003; R-PAC-R, *p* = 0.051; PAC R-post systolic pause-R, *p* < 0.001) except for a P-R interval increase (*p* = 0.032). PAC-associated plethysmography wave amplitudes increased with OAT+ (pre-PAC wave-1, *p* < 0.001; PAC wave-2, *p* = 0.023; post-PAC wave-3, *p* < 0.001).

**Conclusions:**

OAT+ shows promise as an adjunct AF therapy in OSA patients, improving cardiac conduction and vascular function over APAP.

## 1 Introduction

Atrial fibrillation (AF) is highly prevalent in the U.S. and possesses a greater risk in patients with sleep disordered breathing (SDB) vs. patients without SDB (Verrier and Josephson, 2021; Goudis and Ketikoglou, 2017). AF recurrence after catheter ablation is associated with 25% increased risk in patients with obstructive sleep apnea (OSA) (Ng et al., 2011). Mounting evidence implicates repeated hypoxic episodes linked to OSA and central sleep apnea (CSA) acting as chemo-reflex triggers to enhance sympathetic nervous system (SNS) activity responses. Sympathetic over-activity may induce premature atrial contractions (PACs), excitability of cardiac pacemaker and atrial cells, tachycardia and cardiovascular stress. In an animal model, episodes of hypoxia were shown to induce pulmonary vein burst firing and reduction of negative tracheal pressure promptly restored normal sinus rhythm (Goudis and Ketikoglou, 2017).

Pharmacological and ablation therapies are effective for AF, but their efficacy is reduced by OSA. Continuous positive airway pressure (CPAP) treatment for OSA mitigates sympathetic activation, boosts vagal stimulation, and lowers the risk of AF progression and recurrence (Verrier and Josephson, 2021). However, the Sleep Apnea Cardiovascular Endpoints study found that CPAP does not prevent cardiovascular events in patients with moderate-to-severe OSA and cardiovascular disease, with CPAP users showing a non-significant increase in the hazard ratio (1.46) for new-onset AF (McEvoy et al., 2016). Additionally, a large study on adaptive servo-ventilation therapy for CSA indicated increased all-cause and cardiovascular mortality in chronic heart failure patients (Cowie et al., 2015). While CPAP is beneficial in improving blood pressure dipping (Kumagai et al., 2022), reducing the risk of cardiovascular events, enhancing cardiac function and decreasing risks of arrhythmias and pulmonary hypertension in some patients, its effectiveness is limited by poor patient adherence compared with oral appliance (OA) therapy.

OA, which advances the mandible to increase oropharyngeal space and reduce airway collapsibility, is an alternative to CPAP, a first-line treatment for mild to moderate OSA and a second-line option for severe cases or CPAP intolerance (Walsh et al., 2008). Compared to no intervention, OA improves oxygen saturation. A study in severe OSA patients without cardiac disease found OA more effective than CPAP in reducing brain natriuretic peptide levels, suggesting improved cardiac function (Hoekema et al., 2008). Notably, a patient with elevated brain natriuretic peptide levels and AF showed AF improvement with OA. The authors suggested this benefit may be due to reduced breathing effort and intrathoracic pressure, though intra-esophageal pressures, cardiac electrophysiology and hemodynamics were not analyzed.

We hypothesized that PAC temporal changes during sleep in a patient with AF and moderate OSA could reveal differences in cardiac electrophysiology between oral appliance therapy with mouth shield (OAT+) and APAP.

## 2 Report of case

**Figure 1:**
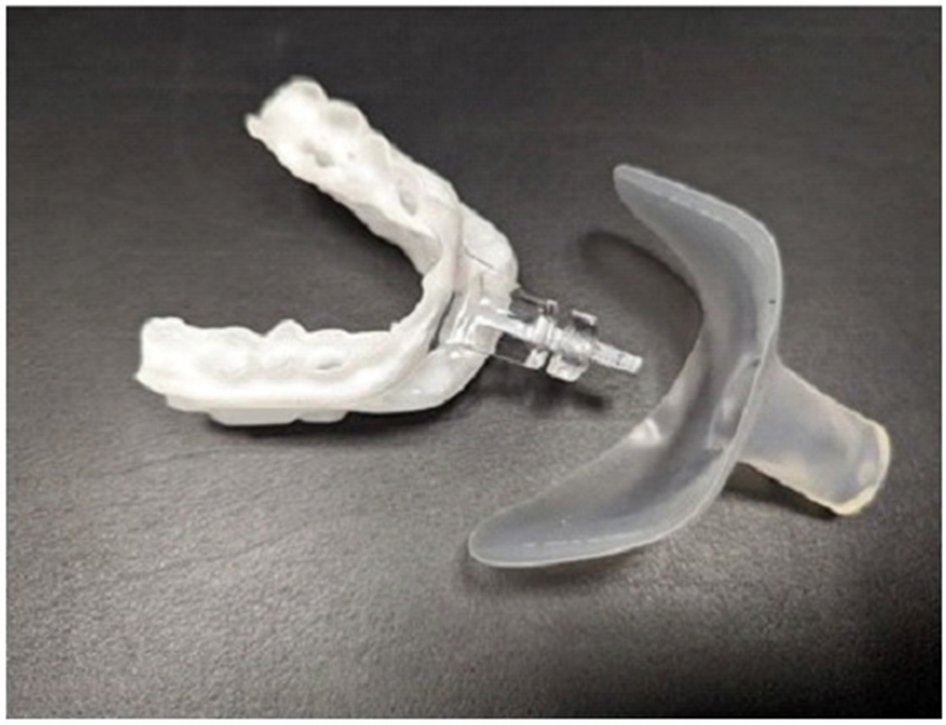
myTAP oral appliance plus mouth shield.

A 67-year-old Caucasian male (Body Mass Index, 27.4 kg/m^2^, 202 lbs) with moderate OSA (Apnea Hypopnea Index, 22 events/hour) and a history of paroxysmal AF managed with three ablations was referred for OAT+. He reported discomfort with APAP therapy using a full-face mask. His only medication is 10 mg daily of Carvedilol extended release for heart rate and blood pressure control. An oral exam revealed no contraindications including Mallampatti score =2, unobstructed nasal breathing (breathing through the nose for 1–2 min with mouth closed, awake and reclined in dental chair), >8 stable teeth per arch to support the OA, healthy gums, jaw joints and muscles. The subject was dentist-fitted with a titratable OAT+ (myTAP, AMI, Carrollton, TX; Figure 1) to reduce upper airway collapse, mouth breathing (MB) and provide comfort (Chakraborty et al., 2010).

## 3 Materials and methods

A home sleep test (HST) evaluated APAP—full-face mask efficacy 12-weeks before the introduction to OAT+ (Table 1). Following a 4-week adjustment period to OAT+, initially titrated to 60% of maximal protrusion, a second HST was performed with OAT+ at this position. Both recordings (>6 h 30 min) collected airflow, respiratory effort, snoring, electrocardiogram (ECG, 2-lead configuration), body position and pulse oximetry with a Nonin finger probe. Electroencephalogram was not recorded. Respiratory dynamics data, including RDI were obtained and analyzed using Noxturnal software and the NOX T3 recorder (NOX Medical, Reykjavík, Iceland). Apnea and hypopnea events were visually scored using revised American Academy of Sleep Medicine 2007 scoring criteria (Berry et al., 2012). RDI was defined as the sum of all apneas and hypopnea events/hour (apneas, >90% reduction in airflow from baseline; hypopneas, 30–90% airflow reduction from baseline associated with ≥3% oxygen desaturation and duration ≥10 seconds). The RDI includes the number of respiratory effort related arousal (RERA) and snore RERA events per hour of recorded time rather than sleep time, so may slightly underestimate the Apnea Hypopnea index (AHI). RERA scoring used ANS arousals based on pulse signal heart rate increases ≥5 beats/min in addition to a sequence of breaths lasting ≥10 s characterized by increasing respiratory effort or by flattening of the inspiratory portion of the nasal pressure (Mayer et al., 2020). Respiratory rate (#breaths/minute), oxygen desaturation index (ODI, #events/h with ≥3% oxygen desaturation), and SaO_2_ (percent), MB (#minutes; ≥3 breaths minimum duration ≥20 dB) and snore percent (snore minutes ≥20 dB/analysis duration minutes) were obtained. The recorder placement followed the manufacturer's recommended mid-thoracic montage.

**Table 1:** Comparison of sleep polygraph variables between OAT+ and APAP.

**Sleep study variable**	**Clinical management**		**% Change**
	**APAP**	**OAT**+	
RDI (#events/h)	2.2	2.7	22.72
Heart rate average (b/m)	51.6	54.8	6.20
Heart rate maximum (b/m)	105	102	−2.94
Oxygen saturation average (%)	94.2	91.5	−2.86
ODI (#events/h)	3.1	2.1	−32.25
Respiratory rate (breaths/m)	12.8	12.4	−3.12
Mouth breathing (#minutes)	0	0	0
Snoring (%)	0	6.2	6.2

RDI, respiratory disturbance index (#events/h); ODI, oxygen desaturation index (#events/h); APAP, auto-adjusting positive airway pressure; OAT+, oral appliance therapy plus mouth shield.

**Figure 2:**
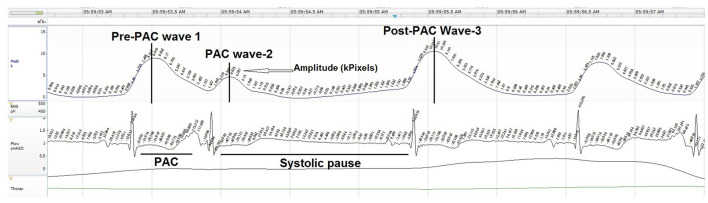
Plethysmography metrics used to identify and quantify time-linked ECG PACs.

Twenty PAC events were randomly selected at similar time points from the start of each night's polygraphy ECG signal (Table 2). PACs were selected with an approximate 20–30 min interval from recording start to potentially capture NREM and REM PAC events. PACs were defined as having a coupling interval to the prior QRS complex ≤ 50% of the mean R-R interval. Manual measures of cardiac conduction and plethysmography, a non-invasive method to evaluate peripheral hemodynamic parameters by measuring changes in peak amplitude (kPixels) variation for each PAC event, with the Noxturnal software (Table 3; Figure 2).

**Table 2:** Comparison of cardiac conduction responses to OAT+ vs. APAP.

**Cardiac conduction variable (s)**	**Clinical management**		**% Change**	***P*-value**
	**APAP**	**OAT**+		
nR-R interval	1.17 [1.08–1.19]	1.09 [1.06–1.12]	−6.67	0.025
Pre-PAC R-R interval	1.16 [1.13–1.20]	1.10 [1.07–1.13]	−5.49	0.003
R-PAC R interval	0.64 [0.61–0.68]	0.60 [0.59–0.63]	−5.37	0.051
PAC R-post systolic pause-R interval	1.64 [1.60–1.68]	1.54 [1.37–1.56]	−6.40	< 0.001
PAC P-R interval	0.032 [0.029–0.035]	0.035 [0.035–0.038]	9.37	0.032
PAC index (#events/h)	48.3	18.4	−61.90	

nR-R, normal R peak to R peak interval; Pre-PAC R-R interval, R-R interval preceding pre-atrial contraction; R-PAC R Interval, R peak to PAC's R peak; PAC R-Post systolic pause- R interval, PAC's R peak including post systolic pause to following R peak; PAC's P wave termination to R peak; APAP, auto-adjusting positive airway pressure; OAT+, oral appliance therapy plus mouth shield [Median [IQR]].

**Table 3:** Comparison of heart rate variability and cardiopulmonary coupling analysis responses to OAT+ vs. APAP.

**Heart rate variability variable**	**Clinical management**		**% Change**	**Dominance**
	**APAP**	**OAT**+		**SNS vs. PNS**
Average RR interval (ms)	1,163	1,064	−8.51	PNS
SDNN (ms)	170	275	61.76	PNS
SDNN index (ms)	160	270	68.75	PNS
RMSSD (ms)	247	455	84.21	PNS
NN50 count	11,072	18,805	69.84	PNS
Percent NN50 of total HR (%)	47.7	84.5	77.14	
SDANN (ms)	43	49	13.95	
Average total power (ms^2^)	8,217	6,875	−16.33	
Average VLF power (ms^2^)	1,226	209	−82.95	
Average LF power (ms^2^)	2,110	654	−69.00	PNS
Average HF power (ms^2^)	2,261	1,241	−45.11	PNS
HRV triangular index	13	34	161.53	
LF/HF ratio	0.93	0.52	−43.52	PNS
**Cardiopulmonary coupling analysis variable**				
HFC	60.8	73.8	21.38	PNS
LFC	24.8	8.1	−67.33	PNS
VLFC	10.5	10.5	0	
e-LFCbb	15.2	4.3	−71.71	PNS
LFC/HFC ratio	0.40	0.10	−73.09	PNS

OAT+, oral appliance therapy plus mouth shield; APAP, automatic positive airway pressure machine; RR Interval, electrocardiogram R peak to R peak time interval; SDNN, standard deviation of normal to normal (NN) heart beat intervals; SDNN index, mean of the standard deviations of all the NN intervals; RMSSD, root mean square of successive differences between normal heartbeats; pNN50, Percentage of successive RR intervals that differ by more than 50 ms; SDANN, Standard deviation of the average NN intervals; VLF band (0.0033–0.04 Hz); LF band (0.04–0.15 Hz); HF or respiratory band (0.15–0.40 Hz); LF/HF ratio, estimates the ratio between sympathetic and parasympathetic nervous system (PNS) activity; HFC, high frequency coupling (0.10 Hz to 0.50 Hz); LFC, low frequency coupling (0.01 Hz to 0.10 Hz); VLFC, very low frequency coupling (0.00391 Hz to 0.010 Hz); e-LFCbb, elevated low frequency coupling broad band (0.006 Hz to 0.10 Hz); LFC/HFC ratio, estimates unstable sleep quality (dominant sympathetic nervous system (SNS) activity).

RemLogic version 1.1 (Embla Systems, Inc., Thornton, CO, USA) software was used to obtain heart rate variability (HRV) and cardiopulmonary coupling (CPC) analyses results from both recordings (Table 3). CPC analysis is an automated method that uses and ECG-derived respiration (EDR) signal directly from QRS axis shifts and is designed to assess breathing dynamics that objectively measures sleep quality in patients with SDB to support our claim that OAT+ increased parasympathetic activity. CPC classification: high frequency coupling (HFC; 0.1–0.5 Hz); low frequency coupling (LFC; 0.01–0.1 Hz); Very low frequency coupling (VLFC; 0.0039–0.01 Hz) (Thomas et al., 2005).

The Mann-Whitney U Test was used for statistical comparison between interventions. The median and interquartile range [IQR] is reported.

## 4 Results

### 4.1 Home sleep test

The HST results showed increases in the RDI (22.7%), average HR (6.2%) and snoring (6.2%) with OAT+ compared with APAP. Decreases in maximum HR (−2.94), oxygen saturation (−2.86%), the oxygen desaturation index (−32.25%), and respiratory rate (−3.12%) with OAT+ compare with APAP was observed. MB was not detected with either intervention (Table 1).

### 4.2 Cardiac conduction

Comparison of cardiac conduction variables between OAT+ and APAP showed OAT+ significantly reduced the duration of the normal R-R interval (p = 0.025), pre-PAC R-R interval (p = 0.003), the PAC R-Post Systolic Pause-R interval (p < 0.001) and a statistical trend for reduction of the R-PAC R interval (p = 0.051), but increased the PAC P-R interval (p = 0.032) compared with APAP. OAT+ decreased the PAC index (#events/h) (−61.9%) compared with APAP (Table 2).

### 4.3 Heart rate variability and cardiopulmonary coupling analysis

HRV: OAT+ decreased the average R-R interval (−8.51%), average total power (−16.33%), average VLF power (−82.95%), average LF power (−69.0%), average HF power (−46.11%) and LF/HF ratio (−43.52%) compared with APAP. LF/HF ratio was lower with OAT+ (0.52) compared with APAP (0.93). OAT+ increased SDNN (61.76%), SDNN index (68.75%), RMSSD (84.21%), NN50 count (69.84%), percent NN50 of total HR (77.14%), SDANN (13.95%), and HRV Triangular index (161.53%) (Table 3).

CPC: OAT+ increased HFC (21.38%) compared with APAP. OAT+ decreased LFC (−67.33%), e-LFCbb (−71.71%) and the LFC/HFC ratio (−73.09%) compared with APAP (Table 3).

### 4.4 Plethysmography

OAT+ significantly increased the pre-PAC wave-1 amplitude (p < 0.001), PAC wave-2 amplitude (p = 0.023) and post-PAC wave-3 amplitude (p < 0.001) compared with APAP (Table 4).

**Table 4:** Comparison of plethysmography wave amplitude responses to OAT+ vs. APAP.

**Plethysmography variable (kPixels)**	**Clinical management**		**% Change**	***P*-value**
	**APAP**	**OAT**+		
Pre-PAC wave-1 Amp	6.14 [5.42–6.82]	9.80 [7.84–11.32]	59.58	< 0.001
PAC wave-2 Amp	3.69 [3.39–4.51]	4.72 [3.91–5.21]	27.95	0.023
Post-PAC wave-3 Amp	6.84 [6.02–8.18]	12.78 [9.59–14.02]	86.63	< 0.001

Pre-PAC wave-1 Amp, pleth wave preceding pre-atrial contraction; PAC wave-2 Amp, pleth wave time-linked to PAC; Post-PAC wave-3 Amp, pleth wave time-linked to post PAC following post systolic pause; APAP, auto-adjusting positive airway pressure; OAT+, oral appliance therapy plus mouth shield [Median [IQR]].

## 5 Discussion

OAT+, combined with a non-selective beta-blocker, shows promise as adjunct therapy for AF patients with OSA, potentially improving cardiac conduction over full-face mask and APAP therapy. Key mechanisms include enhanced nasal airflow, increased parasympathetic activity, higher airway nitric oxide production, and reduced oxygen desaturation index, and lowering of hypoxic triggers.

A recent case report linked AF conversion to normal sinus rhythm and a stable heart rate with optimal CPAP pressure at 9 cm H_2_O, likely due to the reversal of intrathoracic negative pressures causing cardiac stress (Walia et al., 2016). Other factors, such as improved oxygenation and autonomic function, may have contributed, despite mild desaturations. However, conflicting reports question whether CPAP reduces intrathoracic stress as effectively as OA (Schlatzer et al., 2016).

Despite a 2.86% decrease in oxygen saturation, the 32.25% reduction in ODI and 3.12% decrease in respiratory rate, our findings suggest parasympathetic activity increased with OAT+. Our recent study found significant respiratory rate reductions in non-AF patients with mild to severe OSA using OAT+ (Schramm et al., 2024). Improved nasal airflow likely entrains delta and theta brain rhythms, synchronizing cellular networks (Fontanini and Bower, 2006), including the limbic system, which regulates breathing, heart rate, and contraction force via input from the hypothalamus and higher brain regions (Ito et al., 2014; Zelano et al., 2016; Heck et al., 2017).

Our results support the hypothesis that OAT+ reduces cardiovascular stress by mitigating snoring although 6.2% residual snoring persisted without MB. This aligns with previous findings showing OAT+ stabilizes oxygen saturation by promoting nasal breathing and reducing MB over 4–12 weeks (Schramm et al., 2024). Additionally, OA was associated with increased serum nitric oxide and improved endothelial function (Galic et al., 2016). Novel to this report, is the additional HRV and CPC results that support OAT+ increases PNS activity. Low LF/HF ratio (OAT+; 0.52) reflects PNS dominance (Salsone et al., 2018; Shaffer and Ginsberg, 2017). While increased SNS activity can cause shortening of R-R intervals, the irregular nature of AF means that shorter intervals are common due to the increased firing rate of the atria but not necessarily due to increase in SNS activity. In this case, the decrease in R-R interval duration is likely attributed to the reduction in systolic pause duration and lowered PAC index. We speculate these decreases reflect R-R interval normalization and possible reduction in intrathoracic pressure (Schlatzer et al., 2016). Furthermore, the P-R interval increase may reflect Carvedilol's mixed adrenergic blocking effects, which influence cardiac conduction based on sympathetic tone and improve cardiac efficiency by possibly reducing heart rate variability and workload (Cheng et al., 2006). The CPC HFC variable is associated with PNS dominance, stable respiration, blood pressure dipping and non-cyclic alternating patterns in the electroencephalogram (Thomas et al., 2005). The decrease in SNS dominant LFC suggest sympathetic activity down-regulation and the decreased LFC/HFC ratio, demonstrates a shift toward PNS dominance with OAT+.

The findings of this case report should be interpreted with caution because electroencephalogram (EEG) data was not collected in order to make definitive statements about the impact on NREM, REM sleep, and wake and EEG arousals. EEG sleep staging would have allowed us to use the AHI and temporally-link EEG arousals to cardiac events (i.e., increase in HR). Although we used RDI and included RERA and sRERA events, the number of respiratory events occurring during the recording vs. sleep might still be underestimated.

Although we cannot confirm increased NO levels, the rise in plethysmography amplitude likely reflects OAT+-facilitated nasal breathing in addition to Carvedilol's alpha-1 adrenergic blockade, enhancing peripheral vasodilation. These findings align with studies showing reduced arterial stiffness, measured by decreased pulse wave velocity, after 1 year of mandibular advancement device therapy (Lin et al., 2015). Further research is needed to explore OAT+ as adjunct therapy for AF patients co-morbid with OSA.

## Data Availability

The raw data supporting the conclusions of this article will be made available by the authors, without undue reservation.
